# Stigmasterol Protects Against Dexamethasone-Induced Muscle Atrophy by Modulating the FoxO3–MuRF1/MAFbx Signaling Pathway in C2C12 Myotubes and Mouse Skeletal Muscle

**DOI:** 10.3390/biom15111551

**Published:** 2025-11-05

**Authors:** Young-Sool Hah, Seung-Jun Lee, Yeung-Ho Ji, Jeongyun Hwang, Han-Gil Kim, Young-Tae Ju, Jun-Il Yoo, Seung-Jin Kwag

**Affiliations:** 1 Department of Surgery, Institute of Medical Science, Gyeongsang National University College of Medicine and Gyeongsang National University Hospital, Jinju 52727, Republic of Korea; yshah@gnu.ac.kr (Y.-S.H.); drkhg@naver.com (H.-G.K.); drjyt@naver.com (Y.-T.J.); 2 Biomedical Research Institute, Gyeongsang National University Hospital, Jinju 52727, Republic of Korea; 3 Department of Convergence Medical Sciences, Gyeongsang National University, Jinju 52725, Republic of Korea; 0789zxc@gnu.ac.kr (S.-J.L.); dkdl10252@gnu.ac.kr (J.H.); 4 Department of Orthopaedic Surgery, Inha University Hospital, Inha University School of Medicine, Incheon 22332, Republic of Korea; wldudgh503@naver.com

**Keywords:** stigmasterol, muscle atrophy, dexamethasone, C2C12, FoxO3, MuRF1, MAFbx, ubiquitin–proteasome system, mTOR, p70S6K, 4E-BP1, protein synthesis, phytosterol

## Abstract

Glucocorticoid therapy, using agents like dexamethasone (Dexa), often leads to muscle atrophy by increasing protein degradation via the ubiquitin–proteasome system while suppressing protein synthesis. Stigmasterol, a phytosterol with known bioactivities, has an unexplored role in muscle atrophy. This study investigated stigmasterol’s protective effects against Dexa-induced muscle atrophy and its impact on the FoxO3 and mTORC1 signaling pathways. Differentiated C2C12 myotubes were treated with Dexa (50 µM) ± stigmasterol (10 µM), and the morphology, viability, and protein levels in the FoxO3/MuRF1/MAFbx catabolic and mTOR/p70S6K/4E-BP1 anabolic signaling pathways were assessed. C57BL/6 mice received Dexa (20 mg/kg/day i.p.) ± stigmasterol (3 mg/kg/day oral) for 21 days, and the body/muscle mass, bone mineral density (BMD), fiber cross-sectional area (CSA), and muscle protein expression were measured. Stigmasterol (10 µM) was non-toxic and attenuated Dexa-induced reductions in myotube diameter and fusion in vitro, concurrent with suppressing Dexa-induced upregulation of FoxO3/MuRF1/MAFbx proteins and preventing the Dexa-induced dephosphorylation of mTOR/p70S6K/4E-BP1 proteins. In vivo, stigmasterol mitigated Dexa-induced losses in body weight, muscle mass, BMD, and fiber CSA. This protection was associated with attenuated upregulation of FoxO3 and MAFbx proteins in muscle tissue. Stigmasterol protected against Dexa-induced muscle atrophy in vitro and in vivo via modulation of the FoxO3–MAFbx catabolic pathway. These findings suggest stigmasterol inhibits excessive glucocorticoid-induced muscle protein breakdown. It therefore warrants further investigation as a potential therapeutic agent for glucocorticoid myopathy.

## 1. Introduction

Sarcopenia is a syndrome characterized by the progressive, age-related loss of skeletal muscle mass, strength, and function [1,2]. While sarcopenia is typically associated with aging (primary sarcopenia), muscle wasting due to factors such as chronic disease, inflammation, or pharmacological treatments like glucocorticoids is classified as secondary sarcopenia. In this context, dexamethasone-induced muscle atrophy is widely used as a model of secondary sarcopenia, mimicking features of age-related muscle loss, including decreased muscle mass and fiber size [3,4]. Its prevalence increases significantly with age, impacting a substantial portion of the elderly population [5]. Furthermore, sarcopenia is associated with a range of adverse health outcomes, including functional decline, increased risk of falls and fractures, and higher mortality rates, highlighting its significance as a public health concern [2,6]. Muscle mass is regulated by a delicate balance between muscle protein synthesis (anabolism) and muscle protein breakdown (catabolism). Sarcopenia and muscle atrophy resulting from various causes occur when this balance shifts towards excessive breakdown [7].

A major mechanism underlying muscle atrophy is the activation of protein degradation via the ubiquitin–proteasome system (UPS). Playing pivotal roles in this pathway are the muscle-specific E3 ubiquitin ligases, MAFbx (muscle atrophy F-box, also known as Atrogin-1) and MuRF1 (muscle RING finger 1) [8,9]. The expression of these genes is regulated by various upstream signaling pathways. Notably, the Insulin-like Growth Factor-1 (IGF-1)/Akt pathway promotes muscle growth and inhibits protein breakdown. IGF-1/Akt signaling suppresses the transcription of *MAFbx* and *MuRF1* by phosphorylating Forkhead box O (FOXO) family transcription factors (mainly FoxO1 and FoxO3), retaining them in the cytoplasm [10]. Conversely, under atrophic conditions such as starvation, immobilization, inflammation, or excess glucocorticoids, Akt activity is reduced. This allows dephosphorylated FOXO proteins to translocate into the nucleus, where they increase the expression of *MAFbx* and *MuRF1*, thereby promoting protein degradation [11]. Additionally, activation of the energy sensor AMPK (AMP-activated protein kinase) can also influence protein breakdown pathways [12].

Skeletal muscle mass is preserved by the balance between protein synthesis and proteolysis. When this balance is perturbed, muscle atrophy ensues through reduced protein synthesis and increased ubiquitin–proteasome activity [13]. The mTOR (mechanistic target of rapamycin) pathway is a central regulator that is closely related to muscle hypertrophy and atrophy [14]. mTOR forms two biochemically distinct complexes consisting of mTORC1 (mTOR complex 1) and mTORC2 (mTOR complex 2). Of these, mTORC1 integrates amino acids (e.g., leucine via Rag GTPases), growth factors (insulin/IGF-1 via PI3K–Akt), and cellular energy status (AMPK) to control translational output and cell growth [15,16,17]. It promotes protein synthesis by phosphorylating 4E-BP1 (eIF4E-binding protein 1), releasing eIF4E to enable cap-dependent translation initiation [18], and by activating p70S6K (ribosomal protein S6 kinase beta-1), which enhances both translational initiation and elongation, as well as supporting ribosome biogenesis [19]. Through these mechanisms, mTORC1 enhances translational capacity and protein synthesis, sustaining myofiber hypertrophy.

Stigmasterol is a phytosterol (plant sterol) found in various plant sources, including soybeans, nuts, seeds, and several vegetable oils [20,21]. It has been reported to possess diverse biological activities, such as cholesterol-lowering, anti-osteoarthritic, and notably, anti-inflammatory and antioxidant effects [22,23]. Notably, its reported anti-inflammatory and antioxidant activities could be particularly relevant for supporting muscle preservation in catabolic conditions like glucocorticoid myopathy, which are often associated with inflammation and redox stress [20,24,25]. Structurally, stigmasterol is very similar to β-sitosterol, differing primarily by an additional double bond in the side chain. Whereas the structurally related β-sitosterol has been shown to protect against muscle atrophy by modulating MuRF1 and MAFbx expression [26], research on stigmasterol has focused on its metabolic benefits, such as lipid and bile acid regulation, rather than its role in muscle homeostasis [27,28]. Consequently, despite its promising biological activities, no studies have directly investigated the impact of stigmasterol on skeletal muscle mass and function.

Synthetic glucocorticoids, such as dexamethasone, are widely used for treating inflammatory conditions but can cause severe muscle atrophy as a side effect, particularly with long-term or high-dose use [29]. Glucocorticoid-induced muscle atrophy is primarily mediated by increased protein breakdown, specifically through the upregulation of *MAFbx* and *MuRF1* expression via FoxO transcription factors [29]. Despite the various known bioactivities of stigmasterol, its specific impact on muscle mass loss and functional decline, especially under conditions inducing atrophy like glucocorticoid treatment, remains poorly understood. Furthermore, there is a lack of research on its mechanism of action concerning the regulation of muscle protein breakdown pathways.

Therefore, this study aimed to investigate the potential protective effects of stigmasterol against dexamethasone-induced muscle atrophy using both in vitro (C2C12 myotubes) and in vivo (mouse) models. We specifically sought to elucidate the underlying mechanism by examining the impact of stigmasterol on the FoxO3-mediated ubiquitin–proteasome degradation pathway, focusing on the regulation of MuRF1 and MAFbx expression. Concurrently, we assessed the anabolic arm of protein turnover by evaluating mTORC1 signaling via phosphorylation of mTOR, p70S6K, and 4E-BP1.

## 2. Materials and Methods

### 2.1. Chemicals and Reagents

**Figure 1:**
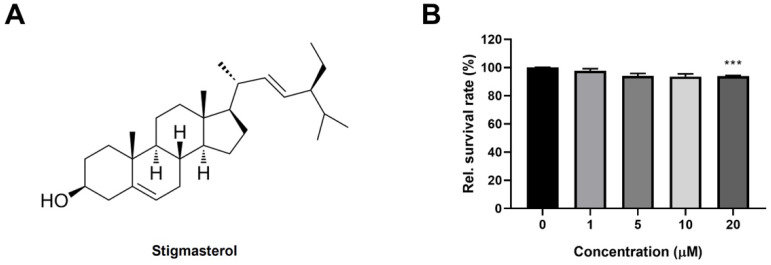
Chemical structure of stigmasterol and its effect on C2C12 myoblast viability. (**A**) Chemical structure of stigmasterol, a plant-derived sterol used in this study. (**B**) Dose–response effect of stigmasterol on C2C12 myoblast viability. Cells were treated with stigmasterol at concentrations of 0, 1, 5, 10, and 20 μM for 24 h, and cell viability was assessed using the CCK-8 assay. Data are presented as mean ± standard deviation (SD) from three independent experiments (*n* = 4). Statistical significance compared to the control (0 μM) is indicated as follows: *** *p* < 0.001 (one-way ANOVA with Tukey’s post hoc test).

Stigmasterol (S0088; Figure 1A) was purchased from Tokyo Chemical Industry (Tokyo, Japan), and dexamethasone (Dexa) was purchased from Sigma-Aldrich (St. Louis, MO, USA). Dulbecco’s Modified Eagle Medium (DMEM), fetal bovine serum (FBS), horse serum (HS), penicillin–streptomycin, and trypsin–EDTA were obtained from Gibco (Thermo Fisher Scientific, Waltham, MA, USA). Giemsa and May–Grunwald staining solutions were sourced from Merck (Darmstadt, Germany). All other chemicals and reagents were of analytical grade and obtained from standard commercial suppliers.

### 2.2. Cell Culture and Treatment

#### 2.2.1. Cell Maintenance and Differentiation

C2C12 mouse myoblast cells (ATCC, Manassas, VA, USA) were cultured in DMEM supplemented with 10% FBS and 1% penicillin–streptomycin at 37 °C in a humidified 5% CO_2_ atmosphere. For differentiation into myotubes, cells were grown to 80% confluence and then switched to differentiation medium (DMEM with 2% HS) for 5 days, with the medium changed every 48 h.

#### 2.2.2. Dose–Response Study

To assess the effect of stigmasterol on C2C12 myoblast viability, cells were seeded in 96-well plates at a density of 5 × 10^3^ cells/well and treated with stigmasterol at concentrations of 0, 1, 5, 10, and 20 μM for 24 h. Subsequently, each well was treated following the protocol provided with the Cell Counting Kit-8 (Dojindo Laboratories, Kumamoto, Japan). Specifically, 10 µL of the CCK-8 reagent was added to each well, and the plate was incubated at 37 °C for 1 h in a humidified incubator with 5% CO_2_. The absorbance at 450 nm, corresponding to the amount of formazan produced by cellular dehydrogenases, was then measured using a microplate reader (Molecular Devices, San Jose, CA, USA). Data were expressed as a percentage of the untreated control.

#### 2.2.3. Dexamethasone-Induced Atrophy and Stigmasterol Treatment

Differentiated C2C12 myotubes were divided into four groups: (1) Control (CTL), treated with vehicle (0.1% DMSO); (2) Dexa, treated with 50 μM dexamethasone; (3) Stigmasterol (S), treated with 10 μM stigmasterol; and (4) Dexa + Stigmasterol (DS), treated with 50 μM dexamethasone and 10 μM stigmasterol. Treatments were applied for 24 h. The concentration of stigmasterol (10 μM) was selected based on the dose–response study (Figure 1B), which showed no significant toxicity at this level.

#### 2.2.4. Giemsa and May–Grunwald Staining

Myotubes were fixed with 4% paraformaldehyde for 15 min and stained with Giemsa and May–Grunwald solutions according to the manufacturer’s instructions. Images were acquired from four randomly selected fields of the stained cells using a camera-equipped microscope (Eclipse 80i, Nikon, Tokyo, Japan). Using NIS Elements software (Version 5.01.00; NIS-Elements Advanced Research, Melville, NY, USA), myotubes within each field were randomly selected to measure their diameter.

#### 2.2.5. Fusion Index Quantification

Myotubes were stained with DAPI (4′,6-diamidino-2-phenylindole) to visualize nuclei. The fusion index was calculated as the percentage of nuclei within multinucleated myotubes (containing ≥3 nuclei) relative to the total number of nuclei, as described previously [30]. At least 10 fields per group were analyzed using a fluorescence microscope (Nikon, Tokyo, Japan).

### 2.3. Cytoplasmic and Nuclear Protein Fractionation

Cytoplasmic and nuclear extracts were prepared using the Rapid, Efficient, and Practical (REAP) method [31]. C2C12 myotubes cultured in 6-well plates were washed with ice-cold PBS (pH 7.4), scraped on ice, and collected into 1.5 mL tubes containing 1 mL ice-cold PBS. The samples were pulse-spun for 10 s in a tabletop microcentrifuge, and the resulting pellets were resuspended in 900 µL ice-cold 0.1% NP-40 in PBS and gently triturated five times. The lysates were centrifuged for 10 s, and 500 µL of the supernatant (cytosolic fraction) was mixed with 100 µL of 6× SDS sample buffer and boiled for 5 min. The remaining supernatant was removed, and the nuclear pellet was washed once with 1 mL of ice-cold 0.1% NP-40/PBS. After discarding the wash supernatant, the pellet was resuspended in 180 µL of 1× SDS sample buffer to obtain the nuclear fraction. All steps were performed on ice. When required, fraction purity was verified by immunoblotting using nuclear (Lamin A/C) and cytosolic (GAPDH) markers.

### 2.4. Western Blot Analysis

C2C12 myotubes and mouse muscle tissues were lysed in RIPA buffer (Thermo Fisher Scientific) supplemented with protease and phosphatase inhibitors (Roche, Basel, Switzerland). Protein concentrations were determined using the BCA Protein Assay Kit (Pierce, Rockford, IL, USA). Equal amounts of protein (30 μg) were separated by 10% SDS-PAGE and transferred to NC membranes (Millipore, Burlington, MA, USA). Membranes were blocked with 5% skim milk in TBST (Tris-buffered saline with 0.1% Tween-20) for 1 h at room temperature, then incubated overnight at 4 °C with primary antibodies against phospho-AMPK (2535S, Cell Signaling Technology, Danvers, MA, USA), AMPK (5831S, Cell Signaling Technology), phospho-FoxO3 (9466S, Cell Signaling Technology), FoxO3 (2497S, Cell Signaling Technology), MuRF1 (sc-398608, Santa Cruz Biotechnology, Dallas, TX, USA), MAFbx (sc-166806, Santa Cruz Biotechnology), Lamin A/C (sc-7292, Santa Cruz Biotechnology), phospho-mTOR (2971S, Cell Signaling Technology), mTOR (2972S, Cell Signaling Technology), phospho-p70S6K (9205S, Cell Signaling Technology), p70S6K (9202S, Cell Signaling Technology), phospho-4E-BP1 (2855S, Cell Signaling Technology), 4E-BP1 (9644S, Cell Signaling Technology), and GAPDH (ATGA0394, ATGen, Seongnam-si, Gyeonggi-do, Republic of Korea). After washing with TBST, membranes were incubated with HRP-conjugated secondary antibodies, including goat anti-mouse IgG (H + L) (31430, Invitrogen, Carlsbad, CA, USA) and goat anti-rabbit IgG (H + L) (31460, Invitrogen) for 1 h at room temperature. Protein bands were visualized using the Clarity Western ECL Substrate (Bio-Rad Laboratories, Inc., Berkeley, CA, USA) and quantified by densitometry using the ChemiDoc^TM^ Touch Imaging System (Bio-Rad Laboratories, Inc.). Expression levels were normalized to GAPDH as a loading control. Original figures can be found in Supplementary Materials.

### 2.5. Animal Model and Experimental Design

#### 2.5.1. Animals

Male C57BL/6 mice with an average body weight of 22 g (6 weeks old) were obtained from Koatech Co., Ltd. (Seoul, Republic of Korea) and housed under controlled conditions (22 ± 2 °C, 12 h light/dark cycle) with ad libitum access to food and water. All experimental procedures were approved by the Institutional Animal Care and Use Committee (IACUC) of Gyeongsang National University (GNU-230719-M0156-01), in accordance with the National Institutes of Health Guide for the Care and Use of Laboratory Animals.

#### 2.5.2. Induction of Muscle Atrophy and Treatment

Mice were randomly divided into three groups (*n* = 8 per group): (1) Control (CTL), receiving vehicle (saline with 0.1% DMSO); (2) Dexa, receiving 20 mg/kg/day dexamethasone i.p.; and (3) Dexa + Stigmasterol (DS), receiving 20 mg/kg/day dexamethasone i.p. and 3 mg/kg/day stigmasterol by oral administration. A stigmasterol-only group was not included in the in vivo experiments based on our preliminary studies, which showed that administration of stigmasterol alone (3 mg/kg/day) did not significantly affect muscle mass or histological parameters in healthy mice. Thus, to minimize animal use and focus on protective effects against dexamethasone-induced atrophy, this group was excluded from the final design. Treatments were administered daily for 21 days. The dose of dexamethasone was selected based on its established ability to induce muscle atrophy in mice [29]. The stigmasterol dose (3 mg/kg/day) was determined based on preliminary studies indicating that this dosage demonstrated efficacy against muscle atrophy indicators while showing no apparent toxicity in the mouse model.

#### 2.5.3. Body Weight and Muscle Mass Measurements

Body weight was recorded weekly throughout the 21-day experimental period. At the end of the experiment, mice were euthanized by CO_2_ inhalation, and the gastrocnemius (GA), tibialis anterior (TA), and extensor digitorum longus (EDL) muscles were dissected and weighed immediately to assess absolute muscle mass. To account for differences in body weight, the wet weight of each muscle was normalized to the final body weight (mg/g). These values are presented as a fold change relative to the control group.

#### 2.5.4. Bone Mineral Density (BMD) Measurement

BMD was measured using a dual-energy X-ray absorptiometry (DXA) scanner (OsteoSys, Seoul, Republic of Korea). Mice were anesthetized with 2% isoflurane during the procedure, and BMD was calculated for the whole body excluding the head.

#### 2.5.5. Immunofluorescence and Cross-Sectional Area (CSA) Analysis

GA and TA muscles were harvested, embedded in OCT compound (Sakura Finetek, Torrance, CA, USA), and snap-frozen in liquid nitrogen. Transverse sections (5 μm) were cut using a cryostat (Leica CM1950; Heidelberg, Germany) and stained with wheat germ agglutinin (WGA) conjugated to Alexa Fluor 488 (W11261; Invitrogen/Thermo Fisher Scientific, Waltham, MA, USA) to visualize muscle fiber boundaries. Images were captured using a fluorescence microscope (Nikon Eclipse ni DSRi2; Nikon, Tokyo, Japan) at 20× magnification. Fiber cross-sectional area (CSA) was quantified using MyoVision (Version 1.0, www.MyoVision.org; University of Kentucky, Lexington, KY, USA) by measuring at least 200 fibers per muscle per mouse [32].

### 2.6. Statistical Analysis

All data are presented as mean ± standard deviation (SD) from at least three independent experiments (for in vitro studies) and as mean ± standard error of the mean (SEM) from eight mice per group (for in vivo studies). Statistical significance was determined using one-way analysis of variance (ANOVA) followed by Tukey’s post hoc test for multiple group comparisons. For the body weight time course, two-way ANOVA with repeated measures was used. *p* < 0.05 was considered statistically significant. Analyses were performed using GraphPad Prism version 9.0 (Version 9.0; GraphPad Software, San Diego, CA, USA).

## 3. Results

### 3.1. Stigmasterol Exhibits Minimal Toxicity on C2C12 Myoblasts at Low Concentrations

To evaluate the potential cytotoxicity of stigmasterol on C2C12 myoblasts, we performed a dose–response study by treating cells with increasing concentrations of stigmasterol (0, 1, 5, 10, and 20 μM) for 24 h (Figure 1B). At concentrations of 1, 5, and 10 μM, stigmasterol had no significant effect on cell viability, with relative survival rates remaining above 95% compared to the untreated control (0 μM). However, at 20 μM, stigmasterol significantly reduced cell viability to 93.8 ± 1.1% (*p* < 0.001), and a more pronounced decrease was observed at higher concentrations. These results indicate that stigmasterol is well-tolerated by C2C12 myoblasts at concentrations up to 10 μM, which was selected for subsequent experiments to ensure minimal toxicity while maximizing potential therapeutic effects.

### 3.2. Stigmasterol Mitigates Dexamethasone-Induced Atrophy in C2C12 Myotubes

**Figure 2:**
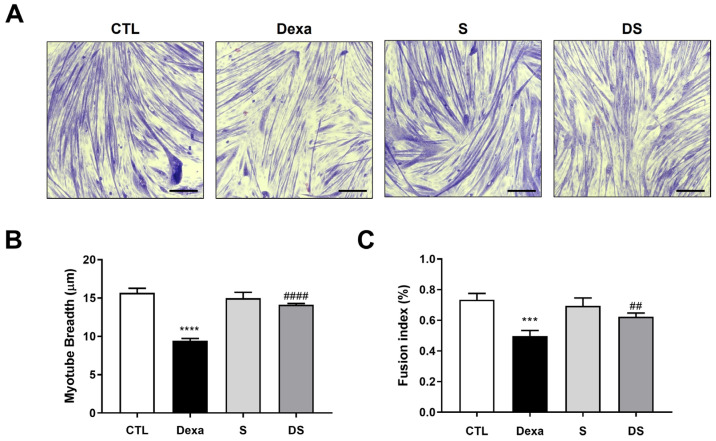
Stigmasterol attenuates dexamethasone-induced atrophy in C2C12 myotubes. (**A**) Representative images of C2C12 myotubes stained with Giemsa and May–Grunwald to assess morphological changes. Myotubes were treated for 24 h under the following conditions: control (CTL, vehicle), dexamethasone (Dexa, 50 μM), stigmasterol (S, 10 μM), or a combination of dexamethasone and stigmasterol (DS, 50 μM Dexa + 10 μM S). Scale bar: 250 μm. (**B**) Quantification of myotube diameter in the four treatment groups. (**C**) Fusion index, calculated as the percentage of nuclei in multinucleated myotubes (≥3 nuclei) relative to the total number of nuclei, reflecting myoblast differentiation and fusion. Data in (**B**,**C**) are presented as mean ± SD from three independent experiments (n = 4). Statistical significance is indicated as follows: *** *p* < 0.001, **** *p* < 0.0001 vs. CTL; ## *p* < 0.01, #### *p* < 0.0001 vs. Dexa (one-way ANOVA with Tukey’s post hoc test). CTL, control; Dexa, dexamethasone; S, stigmasterol; DS, Dexa + stigmasterol.

We next investigated whether stigmasterol could protect C2C12 myotubes from dexamethasone-induced atrophy. Giemsa and May–Grunwald staining revealed significant morphological changes in myotubes treated with 50 μM dexamethasone (Dexa group), including reduced myotube diameter and disrupted structural integrity compared to the control (CTL) group (Figure 2A). Treatment with 10 μM stigmasterol alone (S group) did not alter myotube morphology, while co-treatment with dexamethasone and stigmasterol (DS group) partially restored myotube structure, suggesting a protective effect.

Quantitative analysis of myotube diameter confirmed these observations (Figure 2B). Dexamethasone treatment significantly reduced myotube diameter to 9.4 ± 0.3 μm compared to 15.7 ± 0.6 μm in the control group (**** *p* < 0.0001). Co-treatment with stigmasterol increased the diameter to 14.1 ± 0.2 μm in the DS group (#### *p* < 0.0001 vs. Dexa), indicating that stigmasterol effectively counteracts dexamethasone-induced atrophy. Stigmasterol alone had no significant effect on myotube diameter (15.0 ± 0.8 μm) compared to the control.

We also assessed the fusion index as a measure of myoblast differentiation and fusion (Figure 2C). Dexamethasone treatment reduced the fusion index to 0.50 ± 0.04 compared to 0.73 ± 0.04 in the control group (*** *p* < 0.001). Co-treatment with stigmasterol restored the fusion index to 0.62 ± 0.02 in the DS group (## *p* < 0.01 vs. Dexa), while stigmasterol alone slightly increased the fusion index to 0.69 ± 0.05 (not significant vs. control). These findings suggest that stigmasterol effectively prevents dexamethasone-induced atrophy by preserving the structural integrity and fusion capacity of existing myotubes.

### 3.3. Stigmasterol Downregulates the AMPK–FoxO3–MuRF1-MAFbx Pathway in Dexamethasone-Treated C2C12 Myotubes

**Figure 3:**
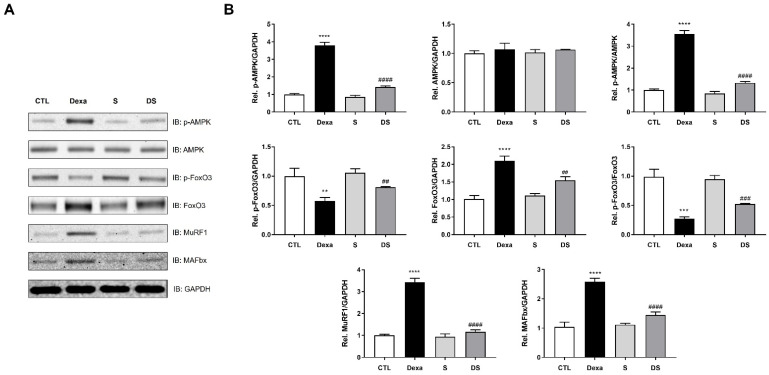
Stigmasterol suppresses the AMPK–FoxO3–MuRF1–MAFbx signaling pathway in dexamethasone-treated C2C12 myotubes. (**A**) Representative Western blot images showing the protein expression of phospho-AMPK (p-AMPK), AMPK, phospho-FoxO3 (p-FoxO3), FoxO3, MuRF1, and MAFbx in C2C12 myotubes. Differentiated myotubes were treated for 24 h with vehicle (CTL), 50 μM dexamethasone (Dexa), 10 μM stigmasterol (S), or a combination of dexamethasone and stigmasterol (DS). GAPDH was used as a loading control. (**B**) The expression levels of p-AMPK, total AMPK, p-FoxO3, total FoxO3, MuRF1, and MAFbx were quantified by densitometry and normalized to GAPDH. The p-AMPK/AMPK and p-FoxO3/FoxO3 ratios were calculated from those normalized values. Data are presented as mean ± SD from three independent experiments. Statistical significance is indicated as follows: ** *p* < 0.01, *** *p* < 0.001, **** *p* < 0.0001 vs. CTL; ## *p* < 0.01, ### *p* < 0.001, #### *p* < 0.0001 vs. Dexa (one-way ANOVA with Tukey’s post hoc test). CTL, control; Dexa, dexamethasone; S, stigmasterol; DS, Dexa + stigmasterol; IB, immunoblot.

To elucidate the upstream molecular mechanism underlying stigmasterol’s protective effects, we first investigated the activation status of AMPK, a key cellular energy sensor, in response to dexamethasone-induced stress. As shown in Figure 3, dexamethasone treatment significantly upregulated the phosphorylation of AMPK (p-AMPK), indicating its activation. Concurrently, the expression of FoxO3, MuRF1, and MAFbx was also significantly increased compared to the control group (**** *p* < 0.0001 for all). Specifically, p-AMPK/AMPK expression increased by 3.55-fold, FoxO3 by 2.07-fold, MuRF1 by 3.41-fold, and MAFbx by 2.49-fold in the Dexa group. Treatment with stigmasterol alone had no significant effect on the expression of these proteins. However, in the DS group, stigmasterol co-treatment significantly abrogated the dexamethasone-induced upregulation of p-AMPK/AMPK (0.37-fold, #### *p* < 0.0001 vs. Dexa) and concurrently suppressed the expression of FoxO3 (0.74-fold, ## *p* < 0.01 vs. Dexa), MuRF1 (0.34-fold, #### *p* < 0.0001 vs. Dexa), and MAFbx (0.56-fold, #### *p* < 0.0001 vs. Dexa). These results indicate that stigmasterol mitigates dexamethasone-induced atrophy by inhibiting the activation of an upstream AMPK-driven signaling cascade that leads to the expression of FoxO3 and its target atrogenes.

### 3.4. Stigmasterol Inhibits the Nuclear Translocation of FoxO3 Protein in Dexamethasone-Treated C2C12 Myotubes

**Figure 4:**
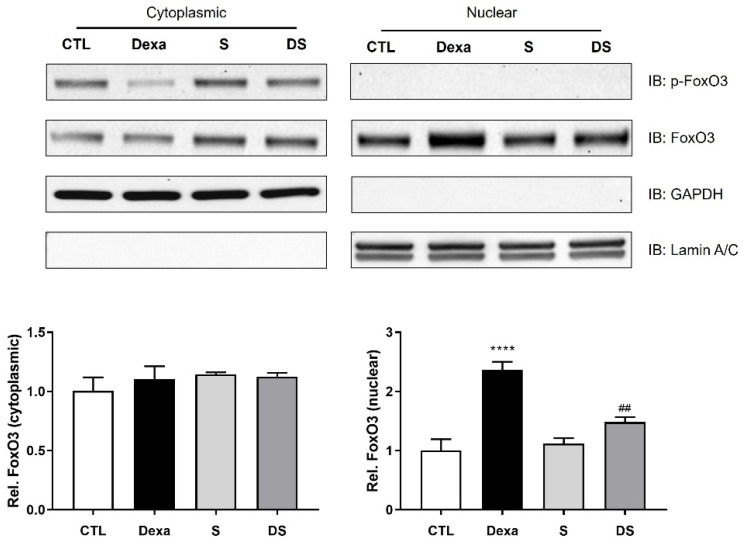
Stigmasterol inhibits the nuclear translocation of FoxO3 in dexamethasone-treated C2C12 myotubes. (**A**) Representative Western blot analysis of FoxO3 protein levels in cytoplasmic and nuclear fractions of C2C12 myotubes. Cells were treated for 24 h with vehicle (CTL), 50 μM dexamethasone (Dexa), 10 μM stigmasterol (S), or a combination (DS). GAPDH and Lamin A/C were used as loading and purity controls for the cytoplasmic and nuclear fractions, respectively. (**B**) Densitometric quantification of FoxO3 protein levels in the cytoplasmic and nuclear fractions. Data are presented as mean ± SD from three independent experiments. Statistical significance is indicated as follows: **** *p* < 0.0001 vs. CTL; ## *p* < 0.01 vs. Dexa (one-way ANOVA with Tukey’s post hoc test). CTL, control; Dexa, dexamethasone; S, stigmasterol; DS, Dexa + stigmasterol; Cytoplasmic, cytoplasmic fraction; Nuclear, nuclear fraction; IB, immunoblot.

Since the transcriptional activity of FoxO3 is critically dependent on its localization within the nucleus, we next examined whether stigmasterol affects the subcellular distribution of FoxO3. Using cellular fractionation, we found that dexamethasone treatment significantly increased the nuclear accumulation of FoxO3 by 2.36-fold compared with the control group (**** *p* < 0.0001), whereas cytosolic FoxO3 levels remained unchanged (Figure 4). In contrast, stigmasterol co-treatment (DS group) markedly attenuated the dexamethasone-induced nuclear translocation of FoxO3, reducing its nuclear abundance by 0.63-fold relative to the Dexa group (## *p* < 0.01). Collectively, these findings provide direct evidence that stigmasterol confers protection against dexamethasone-induced muscle atrophy by inhibiting the nuclear translocation of FoxO3, thereby restricting its ability to activate the transcription of atrophy-related genes.

### 3.5. Stigmasterol Prevents Dexamethasone-Induced Inactivation of the mTOR/p70S6K/4E-BP1 Pathway in C2C12 Myotubes

**Figure 5:**
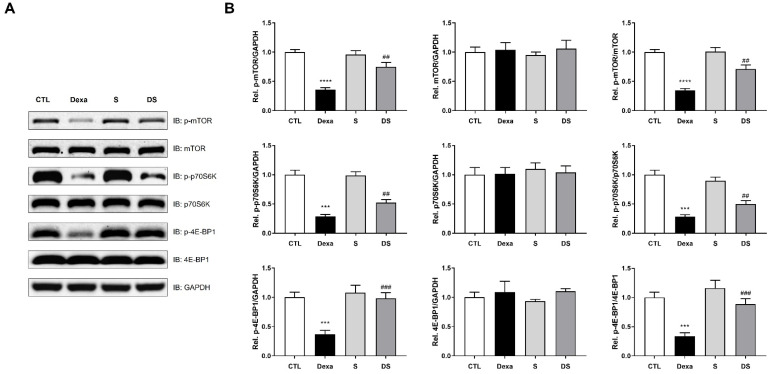
Stigmasterol preserves mTOR/p70S6K/4E-BP1 signaling pathway in dexamethasone-treated C2C12 myotubes. (**A**) Representative Western blot images showing the protein expression of phospho-mTOR (p-mTOR), mTOR, phospho-p70S6K (p-p70S6K), p70S6K, phospho-4E-BP1 (p-4E-BP1), and 4E-BP1 in C2C12 myotubes. Differentiated myotubes were treated for 24 h with vehicle (CTL), 50 μM dexamethasone (Dexa), 10 μM stigmasterol (S), or a combination of dexamethasone and stigmasterol (DS). GAPDH was used as a loading control. (**B**) The expression levels of p-mTOR, total mTOR, p-p70S6K, total p70S6K, p-4E-BP1, and total 4E-BP1 were quantified by densitometry and normalized to GAPDH. The p-mTOR/mTOR, p-p70S6K/p70S6K, and p-4E-BP1/4E-BP1 ratios were calculated from those normalized values. Data are presented as mean ± SD from three independent experiments. Statistical significance is indicated as follows: *** *p* < 0.001, **** *p* < 0.0001 vs. CTL; ## *p* < 0.01, ### *p* < 0.001 vs. Dexa (one-way ANOVA with Tukey’s post hoc test). CTL, control; Dexa, dexamethasone; S, stigmasterol; DS, Dexa + stigmasterol; IB, immunoblot.

To investigate the effect of stigmasterol on protein synthesis, we assessed the mTORC1 signaling pathway, an integrator of nutrient and growth factor signals, in dexamethasone-treated C2C12 myotubes. As shown in Figure 5, dexamethasone treatment significantly downregulated the phosphorylation of mTOR (p-mTOR), indicating its inactivation. Concurrently, the expression of phosphorylated p70S6K (p-p70S6K) and phosphorylated 4E-BP1 (p-4E-BP1) was also significantly decreased compared to the control group (*** *p* < 0.001 for p-p70S6K and p-4E-BP1, **** *p* < 0.0001 for p-mTOR). Specifically, p-mTOR/mTOR ratio decreased by 0.35-fold, p-p70S6K/p70S6K by 0.28-fold, and p-4E-BP1/4E-BP1 by 0.34-fold in the Dexa group. Treatment with stigmasterol alone had no significant effect on the expression of these proteins. However, in the DS group, stigmasterol co-treatment significantly abrogated the dexamethasone-induced downregulation of p-mTOR/mTOR (2.05-fold, ## *p* < 0.01 vs. Dexa) and concurrently suppressed the expression of p-p70S6K/p70S6K (1.77-fold, ## *p* < 0.01 vs. Dexa) and p-4E-BP1/4E-BP1 (2.62-fold, ### *p* < 0.001 vs. Dexa). These results indicate that stigmasterol counteracts Dexa-induced catabolic signaling by restoring mTORC1 activity, as evidenced by the renewed phosphorylation of p70S6K and 4E-BP1, which relieves cap-dependent translational repression and prevents myofiber atrophy.

### 3.6. Stigmasterol Prevents Dexamethasone-Induced Muscle Atrophy in Mice

**Figure 6:**
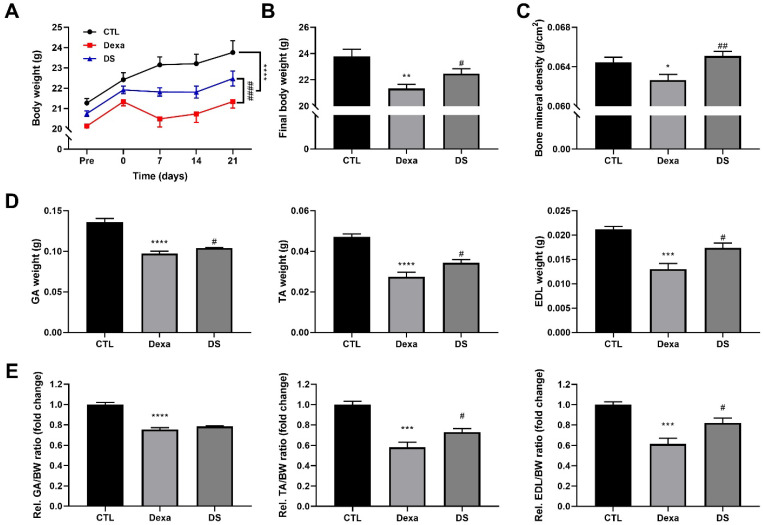
Stigmasterol ameliorates dexamethasone-induced muscle atrophy in mice. (**A**) Body weight changes over 21 days in mice treated with vehicle (CTL), dexamethasone (Dexa, 20 mg/kg/day), stigmasterol (S, 3 mg/kg/day), or a combination of dexamethasone and stigmasterol (DS, 20 mg/kg/day Dexa + 3 mg/kg/day S). (**B**) Final body weight at day 21. (**C**) Bone mineral density (BMD) measured by dual-energy X-ray absorptiometry (DXA). (**D**) Absolute muscle mass of the gastrocnemius (GA), tibialis anterior (TA), and extensor digitorum longus (EDL) muscles at day 21. (**E**) Relative muscle weight-to-body weight (BW) ratios for GA, TA, and EDL, expressed as a fold change. Data in (**A**–**E**) are presented as mean ± standard error of the mean (SEM) (*n* = 8 mice per group). Statistical significance is indicated as follows: * *p* < 0.05, ** *p* < 0.01, *** *p* < 0.001, **** *p* < 0.0001 vs. CTL; # *p* < 0.05, ## *p* < 0.01, #### *p* < 0.0001 vs. Dexa (two-way ANOVA with repeated measures for (**A**); one-way ANOVA with Tukey’s post hoc test for (**B**–**E**)). CTL, control; Dexa, dexamethasone; S, stigmasterol; DS, Dexa + stigmasterol; GA, gastrocnemius; TA, tibialis anterior; EDL, extensor digitorum longus.

We further evaluated stigmasterol’s protective effects in a mouse model of dexamethasone-induced muscle atrophy. Body weight was monitored over 21 days (Figure 6A). The Dexa group exhibited a significant reduction in body weight starting at day 7, reaching 21.2 ± 0.9 g by day 21 compared to 23.8 ± 1.4 g in the control group (**** *p* < 0.0001). Co-treatment with stigmasterol (DS group) attenuated this weight loss, with a final body weight of 22.5 ± 1.0 g (#### *p* < 0.0001 vs. Dexa).

Final body weight measurements at day 21 (Figure 6B) confirmed these trends, with the DS group showing a significant improvement over the Dexa group (# *p* < 0.05). Bone mineral density (BMD) was also assessed (Figure 6C). Dexamethasone treatment reduced BMD to 0.063 ± 0.002 g/cm^2^ compared to 0.064 ± 0.001 g/cm^2^ in the control group (* *p* < 0.05). Co-treatment with stigmasterol restored BMD to 0.065 ± 0.001 g/cm^2^ in the DS group (## *p* < 0.01 vs. Dexa).

Muscle mass of the gastrocnemius (GA), tibialis anterior (TA), and extensor digitorum longus (EDL) muscles was measured (Figure 6D). Dexamethasone significantly reduced muscle mass in all three muscles (GA: 0.10 ± 0.01 g, TA: 0.03 ± 0.007 g, EDL: 0.013 ± 0.003 g) compared to the control group (GA: 0.14 ± 0.01 g, TA: 0.05 ± 0.004 g, EDL: 0.021 ± 0.001 g; *** *p* < 0.001 for EDL, **** *p* < 0.0001 for GA and TA vs. CTL). Co-treatment with stigmasterol increased muscle mass in the DS group (TA: 0.034 ± 0.005 g, EDL: 0.017 ± 0.003 g; # *p* < 0.05 vs. Dexa). To determine if the loss of muscle mass was disproportionate to the change in total body weight, we also analyzed the muscle weight-to-body weight ratios (Figure 6E). Similar to the absolute mass measurements, the relative weights of the GA, TA, and EDL muscles were significantly reduced in the Dexa group compared to the CTL group (GA: 0.753 ± 0.020-fold, TA: 0.582 ± 0.049-fold, and EDL: 0.614 ± 0.054-fold; *** *p* < 0.001 for TA and EDL, **** *p* < 0.0001 for GA). Co-treatment with stigmasterol increased the muscle weight-to-body weight ratios in the DS group (TA: 0.728 ± 0.034-fold and EDL: 0.820 ± 0.047-fold; # *p* < 0.05 vs. Dexa). These results demonstrate that stigmasterol effectively prevents dexamethasone-induced muscle loss and preserves BMD in vivo.

### 3.7. Stigmasterol Restores Muscle Fiber Cross-Sectional Area in Dexamethasone-Treated Mice

**Figure 7:**
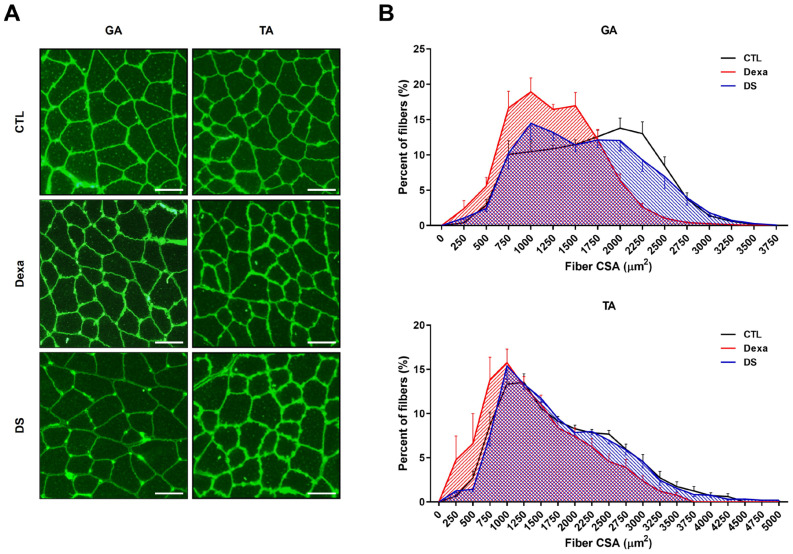
Stigmasterol preserves muscle fiber cross-sectional area in dexamethasone-treated mice. (**A**) Representative immunofluorescence images of gastrocnemius (GA) and tibialis anterior (TA) muscle cross-sections stained with wheat germ agglutinin (WGA) to visualize fiber boundaries. Mice were treated for 21 days with vehicle (CTL), dexamethasone (Dexa, 20 mg/kg/day), stigmasterol (S, 3 mg/kg/day), or a combination of dexamethasone and stigmasterol (DS, 20 mg/kg/day Dexa + 3 mg/kg/day S). Scale bar: 50 μm. (**B**) Histogram distribution of fiber cross-sectional area (CSA) in GA and TA muscles, showing the percentage of fibers within specified CSA ranges. Data are presented as mean ± SEM (*n* = 8 mice per group). CTL, control; Dexa, dexamethasone; S, stigmasterol; DS, Dexa + stigmasterol; GA, gastrocnemius; TA, tibialis anterior.

To further assess stigmasterol’s impact on muscle morphology, we analyzed the cross-sectional area (CSA) of muscle fibers in the GA and TA muscles using immunofluorescence (Figure 7). Dexamethasone treatment significantly reduced fiber CSA in both muscles (GA: 1258 ± 176 μm^2^, TA: 1431 ± 380 μm^2^) compared to the control group (GA: 1677 ± 197 μm^2^, TA: 1786 ± 286 μm^2^*. Co-treatment with stigmasterol increased fiber CSA in the DS group (GA: 1607 ± 343 μm^2^, TA: 1781 ± 258 μm^2^). Histogram analysis of fiber CSA distribution confirmed that stigmasterol shifted the distribution toward larger fiber sizes in the DS group compared to the Dexa group. These findings indicate that stigmasterol preserves muscle fiber size in the presence of dexamethasone, further supporting its protective role against muscle atrophy.

### 3.8. Stigmasterol Reduces Expression of Atrophy-Related Proteins in Mouse Muscle Tissues

**Figure 8:**
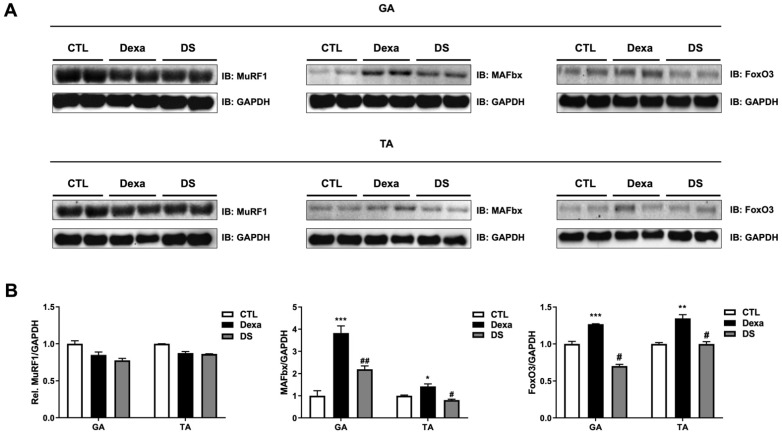
Stigmasterol reduces expression of atrophy-related proteins in mouse muscle tissues. (**A**) Western blot analysis of MuRF1, MAFbx, and FoxO3 protein expression in gastrocnemius (GA) and tibialis anterior (TA) muscles from mice treated for 21 days with vehicle (CTL), dexamethasone (Dexa, 20 mg/kg/day), stigmasterol (S, 3 mg/kg/day), or a combination of dexamethasone and stigmasterol (DS, 20 mg/kg/day Dexa + 3 mg/kg/day S). GAPDH was used as a loading control. (**B**) Densitometric quantification of MuRF1, MAFbx, and FoxO3 expression levels, normalized to GAPDH. Data are presented as mean ± SEM (*n* = 8 mice per group). Statistical significance is indicated as follows: * *p* < 0.05, ** *p* < 0.01, *** *p* < 0.001 vs. CTL; # *p* < 0.05, ## *p* < 0.01 vs. Dexa (one-way ANOVA with Tukey’s post hoc test). CTL, control; Dexa, dexamethasone; S, stigmasterol; DS, Dexa + stigmasterol; GA, gastrocnemius; TA, tibialis anterior; IB, immunoblot.

Western blot analysis of GA and TA muscle tissues revealed that dexamethasone significantly upregulated the expression of MAFbx and FoxO3 in both muscles compared to the control group (Figure 8). In the GA muscle, MuRF1 expression decreased to 0.85-fold, whereas MAFbx expression increased by 3.82-fold and FoxO3 by 1.26-fold in the Dexa group. Similar trends were observed in the TA muscle (MuRF1: 0.87-fold, MAFbx: 1.42-fold, FoxO3: 1.34-fold). Co-treatment with stigmasterol in the DS group significantly reduced these levels (GA: MuRF1 0.92-fold, MAFbx 0.57-fold, FoxO3 0.55-fold; TA: MuRF1 0.98-fold, MAFbx 0.56-fold, FoxO3 0.74-fold; * *p* < 0.05, ** *p* < 0.01, and *** *p* < 0.001 vs. CTL; # *p* < 0.05 vs. Dexa). These results confirm that stigmasterol’s protective effects in vivo are mediated by the downregulation of the FoxO3–MAFbx pathway, consistent with our in vitro findings.

## 4. Discussion

This study provides a definitive molecular mechanism for the protective effects of stigmasterol against dexamethasone (Dexa)-induced skeletal muscle atrophy. Our most significant finding is that stigmasterol abrogates Dexa-induced catabolic signaling by inhibiting the activation of the energy sensor AMPK, which in turn prevents the nuclear translocation of the key transcription factor FoxO3 (Figure 3 and Figure 4). This upstream regulation directly leads to the suppression of the E3 ubiquitin ligases MuRF1 and MAFbx. In parallel with this anti-catabolic action, stigmasterol preserves the anabolic arm of protein turnover by restoring mTOR/p70S6K/4E-BP1 signaling, which maintains protein accretion and prevents Dexa-induced losses in muscle mass (Figure 5). This newly identified mechanism explains the robust preservation of myotube morphology in vitro and the mitigation of muscle mass loss in vivo (Figure 2, Figure 6, Figure 7 and Figure 8).

Mechanistically, the additional experiments introduced here provide direct readouts of FoxO3 activity and localization. First, Dexa increased p-AMPK in myotubes and upregulated FoxO3, MuRF1, and MAFbx; however, stigmasterol co-treatment significantly blunted these Dexa effects (Figure 3). Second, cytoplasmic/nuclear fractionation showed that Dexa markedly enhanced nuclear accumulation of FoxO3 in C2C12 myotubes, whereas stigmasterol reduced Dexa-induced FoxO3 nuclear localization (with p-FOXO3 and total FOXO3 examined in the respective fractions) (Figure 4). These data indicate that stigmasterol limits FoxO3 transcriptional activity by restraining its nuclear presence, a mechanism consistent with the established control of FoxO factors through phosphorylation-dependent cytosolic sequestration [33].

In vivo, Dexa significantly increased MAFbx and FoxO3 in gastrocnemius (GA) and tibialis anterior (TA) muscles, and stigmasterol lowered both toward control levels (Figure 8). Notably, MuRF1 did not increase with Dexa at the 21-day time point, highlighting temporal and tissue-context dependencies; nevertheless, stigmasterol consistently attenuated the Dexa-driven FoxO3–MAFbx axis in this model (Figure 8). These observations, together with the in vitro findings, support a central role for FoxO3–MAFbx modulation in stigmasterol’s protective action.

The present results align with the well-characterized role of glucocorticoids in activating the UPS and FoxO transcription factors to induce MuRF1 and MAFbx, promoting muscle protein breakdown [29,33]. A critical question arising from our findings is the upstream pharmacological mechanism by which stigmasterol exerts its action. Our data strongly support targeting intracellular signaling pathways rather than direct chemical neutralization of dexamethasone, for which there is no evidence. While our study did not identify the primary molecular target, we can propose two plausible, literature-based hypotheses. First, stigmasterol could potentially modulate the activity of the glucocorticoid receptor (GR) itself, for instance, by interfering with ligand binding or nuclear translocation, thereby blunting the initial genomic signal. Second, and more directly related to our findings, stigmasterol may act by reducing the initial cellular stress that triggers AMPK activation. Our finding that stigmasterol blocks Dexa-induced AMPK activation is highly significant. It is well-established in the literature that glucocorticoids can provoke mitochondrial dysfunction and cellular energy deficits, which are known upstream activators of AMPK [34]. In fact, emerging evidence strongly suggests that such mitochondrial dysfunction is not merely a consequence of atrophy but is a primary causal event that triggers catabolic signaling [35,36]. As a crucial sensor of cellular energy status, AMPK is activated by mitochondrial stress and, in turn, can help coordinate the cellular response to restore mitochondrial health and homeostasis [37,38]. Therefore, a plausible mechanism is that stigmasterol interferes with these initial stress signals, thereby preventing the subsequent engagement of the AMPK–FOXO3 atrophy program that we have demonstrated. In our study, Dexa increased p-AMPK, and co-treatment with stigmasterol reduced this response, parallel to decreased FoxO3 nuclear localization and reduced atrogene expression, consistent with the dampening of AMPK–FOXO3 signaling. AMPK has also been implicated in disuse-induced wasting in vivo, where AMPK activity facilitates FoxO3-dependent proteolysis; genetic down-modulation of AMPK attenuates unloading atrophy and blunts proteolytic signaling [39]. Collectively, our data and the literature support a model in which stigmasterol counteracts Dexa-triggered energy-stress signaling to restrain FoxO3 activity and UPS-mediated proteolysis.

**Figure 9:**
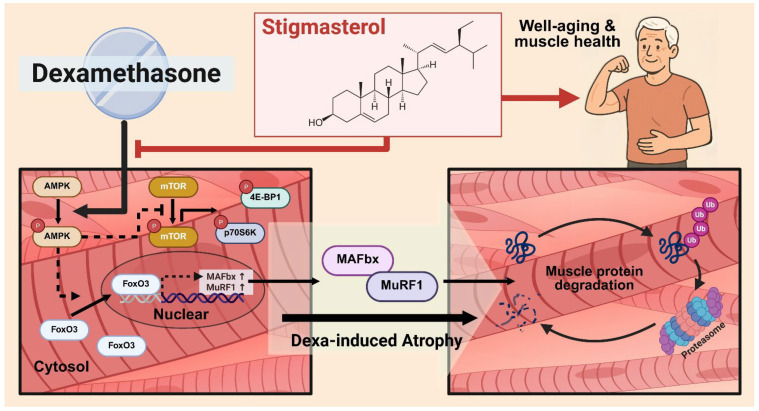
Working model for the protective action of stigmasterol against Dexa-induced muscle atrophy. The schematic integrates the present findings. Dexamethasone (Dexa) activates AMPK (↑p-AMPK) and promotes FOXO3 nuclear translocation, which increases the expression of the E3 ubiquitin ligases MAFbx (and, in vitro, MuRF1) and thereby enhances the capacity for ubiquitin–proteasome–mediated protein degradation. Concurrently, Dexa suppresses the anabolic mTORC1 axis (↓p-mTOR, ↓p-p70S6K, ↓p-4E-BP1), reducing translational capacity and contributing to muscle atrophy. Stigmasterol attenuates AMPK phosphorylation, reduces FOXO3 nuclear localization, downregulates FOXO3 targets, and restores mTORC1 signaling. Together, these anti-catabolic and pro-anabolic effects preserve muscle mass and fiber size. The chemical structure of stigmasterol and a conceptual outcome (muscle health) are shown for context. The model is based on data in Figure 2, Figure 3, Figure 4, Figure 5, Figure 6, Figure 7 and Figure 8 (p-AMPK, atrogene, p-mTOR, p-p70S6K, and p-4E-BP1 changes in myotubes; FOXO3 nuclear localization; and in vivo changes in FOXO3/MAFbx together with muscle mass and CSA).

In parallel with its anti-catabolic effects on the AMPK–FOXO3–atrogene axis, stigmasterol preserves the anabolic arm of protein turnover by restoring mTORC1 signaling in Dexa-treated C2C12 myotubes. In our experiments, Dexa decreased the phosphorylation of mTOR, p70S6K, and 4E-BP1, consistent with the suppression of cap-dependent translation and reduced ribosome biogenesis. However, co-treatment with stigmasterol reversed these effects (Figure 5), indicating renewed mTORC1 output that supports translational initiation/elongation, as well as net protein accretion. This pro-anabolic rescue complements the anti-catabolic action, providing a mechanistic basis for the preserved myotube morphology in vitro. The mTORC1 findings align with the AMPK data, as AMPK inhibits mTORC1 by phosphorylating TSC2 and Raptor, restraining translation under energetic stress [40]. By dampening Dexa-induced AMPK activation, stigmasterol would be expected to disinhibit mTORC1, allowing phosphorylation of p70S6K and 4E-BP1 to resume and relieving the brake on cap-dependent initiation. Together, these results support a dual-axis model in which stigmasterol limits proteolysis (via FOXO3/MAFbx/MuRF1) while sustaining protein synthesis (via mTOR/p70S6K/4E-BP1), stabilizing muscle protein balance during glucocorticoid stress. A schematic integrating these findings is shown in Figure 9. Integrating our new signaling data, Figure 9 summarizes how stigmasterol blunts Dexa-induced atrophy: Dexa elevates p-AMPK, drives FOXO3 nuclear accumulation, and upregulates its E3 ligase targets (MAFbx and, in vitro, MuRF1), converging on ubiquitin–proteasome-mediated protein breakdown. Concordant with AMPK inhibition of mTORC1, Dexa simultaneously suppresses the anabolic mTORC1 axis (in vitro, mTOR/p70S6K/4E-BP1). Stigmasterol attenuates AMPK phosphorylation, reduces FOXO3 nuclear localization, and lowers FOXO3-target expression while restoring mTORC1 signaling, thereby diminishing the catabolic drive and preserving muscle morphology and mass in vitro and in vivo.

A key finding of this study is the apparent divergence in MuRF1 regulation between our in vitro and in vivo models. While Dexa robustly induced MuRF1 in myotubes, this effect was absent in muscle at the 21-day time point. This is not a conflicting result but rather reflects the known complex and differential regulation of atrogenes. Specifically, *MuRF1* expression is known to be an early and transient event in response to catabolic stimuli, while *MAFbx* upregulation can be more sustained. Our 21-day endpoint therefore likely captures the chronic state where the FoxO3–MAFbx axis remains dominant, a temporal distinction that is authoritatively discussed in the literature [41]. This is further supported by literature demonstrating that MuRF1 and MAFbx are not functionally redundant and are subject to distinct regulatory inputs in a complex in vivo systemic environment compared to a simplified cell culture model [42]. Thus, our data accurately reveal that in a model of chronic glucocorticoid excess, stigmasterol’s protective effect is most strongly associated with the sustained suppression of the FoxO3–MAFbx signaling axis.

It is also important to interpret UPS marker changes in the broader context of proteostasis. Upregulation of E3 ligases and protein ubiquitination reflects increased catabolic drive, but these readouts are not invariably equivalent to instantaneous proteasomal degradation rates, and ubiquitin signals can be non-degradative [43,44]. Within this dynamic framework, stigmasterol’s consistent downregulation of FoxO3 and MAFbx across systems, together with reduced FoxO3 nuclear localization, indicates a significant reduction in catabolic signaling pressure that aligns with the morphological and mass-preserving outcomes observed.

The present study now includes direct measures of FoxO3 activation state (p-FOXO3) and subcellular localization, strengthening the mechanistic link between stigmasterol and FoxO3 signaling. However, this study has several limitations. While our data robustly demonstrate a protective effect on overall muscle fiber cross-sectional area, we did not investigate qualitative changes in fiber composition. Given that glucocorticoids preferentially target fast-twitch type IIb fibers, an analysis of MyHC-IIb expression would provide more specific insight into the protective mechanism. Similarly, an ultrastructural analysis via TEM focused on the neuromuscular junction and mitochondria, while valuable, extends beyond the primary scope of this study, which was to define the role of stigmasterol in modulating the AMPK–FoxO3–atrogene signaling axis. These remain important avenues for future work. Furthermore, we did not quantify Akt phosphorylation, which would help define whether stigmasterol also preserves the canonical IGF-1/Akt brake on FoxO3 [33]. Genetic perturbation studies (e.g., *FoxO3* knockdown/overexpression) and pharmacologic dissection of AMPK and Akt pathways would further refine causality. In vivo, the addition of a stigmasterol-only group would allow for a direct assessment of baseline effects. Our rationale for exclusion (pilot data indicating a minimal independent effect at 3 mg/kg/day) is provided in the Section 2; however, confirmation in the main experiment would be ideal. Finally, functional readouts (strength, endurance) were not measured and should be considered for future studies alongside mass and histological endpoints.

In summary, stigmasterol counters Dexa-induced muscle atrophy by suppressing the FoxO3–MAFbx catabolic program, as evidenced by reduced FoxO3 nuclear localization and attenuation of Dexa-induced p-AMPK in myotubes, accompanied by concordant decreases in MAFbx and FoxO3 in vivo. In parallel, it preserves mTORC1-dependent protein synthesis by restoring phosphorylation of mTOR, p70S6K, and 4E-BP1 in myotubes. These findings, together with prior work on phytosterols, suggest that stigmasterol is a promising candidate for mitigating glucocorticoid myopathy and warrant further mechanistic and translational evaluation.

## 5. Conclusions

This study provides compelling evidence that stigmasterol effectively ameliorates dexamethasone-induced muscle atrophy in vitro and in vivo. We have elucidated a definitive molecular mechanism for this protection, demonstrating that stigmasterol acts by inhibiting the dexamethasone-induced activation of the AMPK stress-signaling pathway. This upstream blockade prevents the nuclear translocation of the transcription factor FoxO3, thereby suppressing the downstream expression of the catabolic E3 ubiquitin ligases MuRF1 and MAFbx. Concurrently, stigmasterol preserves the anabolic arm of protein turnover by restoring mTORC1 signaling, as reflected by renewed phosphorylation of mTOR, p70S6K, and 4E-BP1. These findings highlight stigmasterol as a promising natural compound for combating muscle wasting, particularly associated with glucocorticoid use, and strongly warrant further investigation into its broader therapeutic and preventative potential for maintaining muscle health.

## Data Availability

The data presented in this study are available within the article or Supplementary Materials.

## Supplementary Materials

The following supporting information can be downloaded at https://www.mdpi.com/article/10.3390/biom15111551/s1. File S1. Uncropped western blots cited in Figures.
