# Supplementary material for: Stigmasterol Protects Against Dexamethasone-Induced Muscle Atrophy by Modulating the FoxO3–MuRF1/MAFbx Signaling Pathway in C2C12 Myotubes and Mouse Skeletal Muscle
Source: Biomolecules. 2025 Nov 5;15(11):1551. doi: 10.3390/biom15111551 (PMC12650398; doi:10.3390/biom15111551)
Supplement: Supplementary file 1 [file biomolecules-15-01551-s001.zip › biomolecules-3873098-supplementary.pptx]

## Slide 1
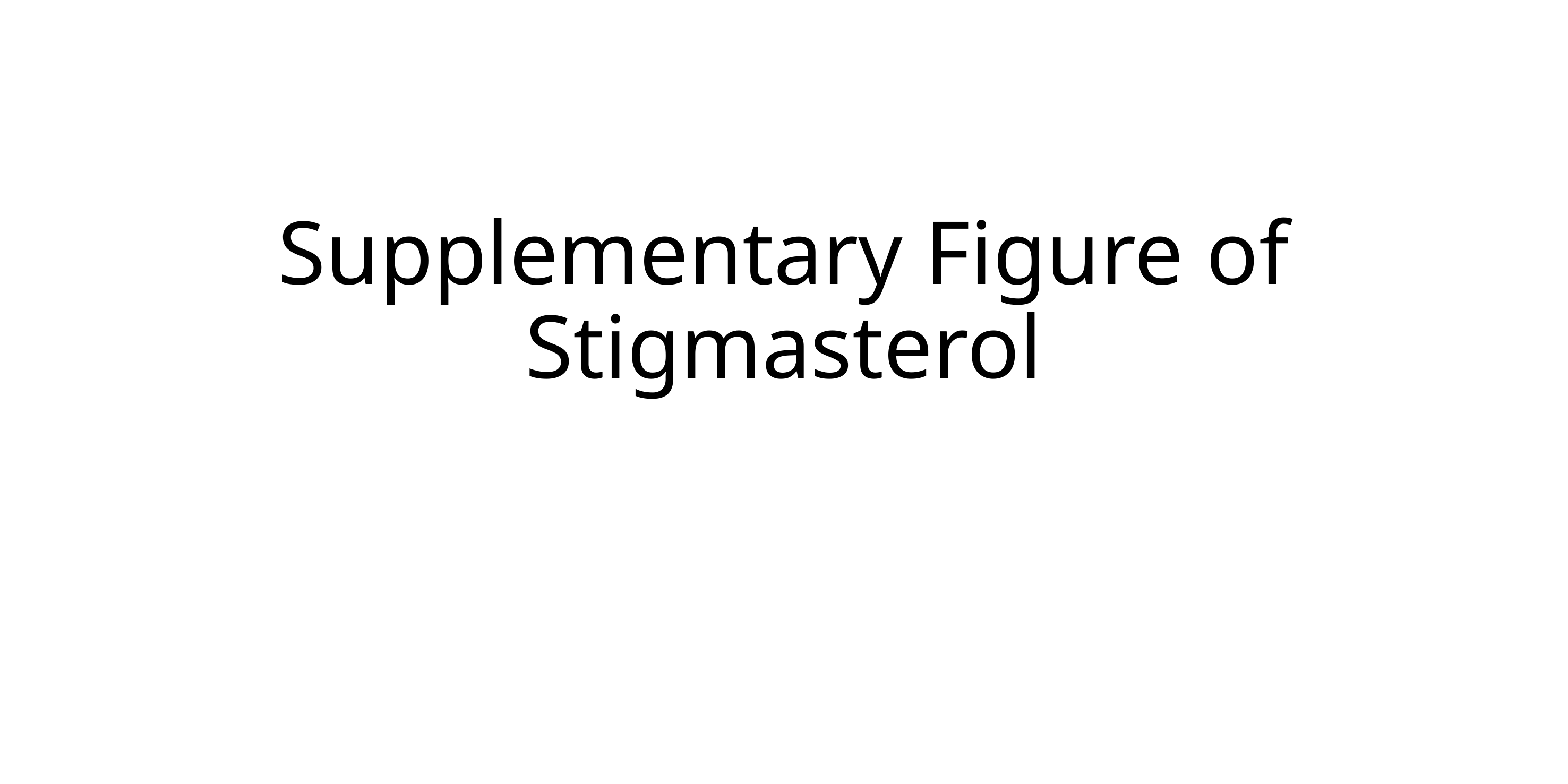

# Supplementary Figure of Stigmasterol

## Slide 2
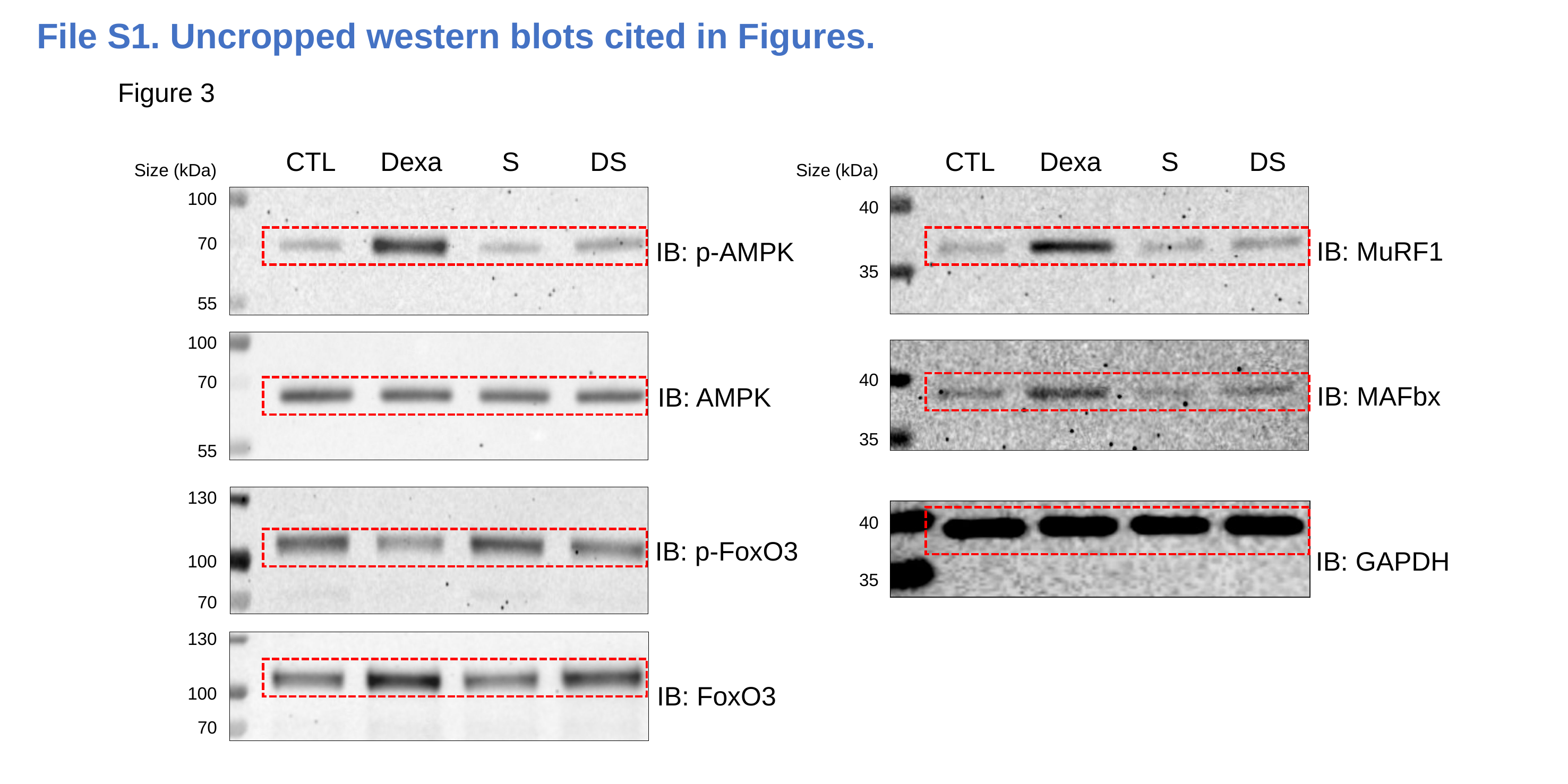

File S1. Uncropped western blots cited in Figures.
Figure 3
CTL
CTL
Dexa
S
DS
Dexa
S
DS
Size (kDa)
Size (kDa)
100
40
70
IB: MuRF1
IB: p-AMPK
35
55
100
40
70
IB: MAFbx
IB: AMPK
35
55
130
40
IB: p-FoxO3
IB: GAPDH
100
35
70
130
IB: FoxO3
100
70

## Slide 3
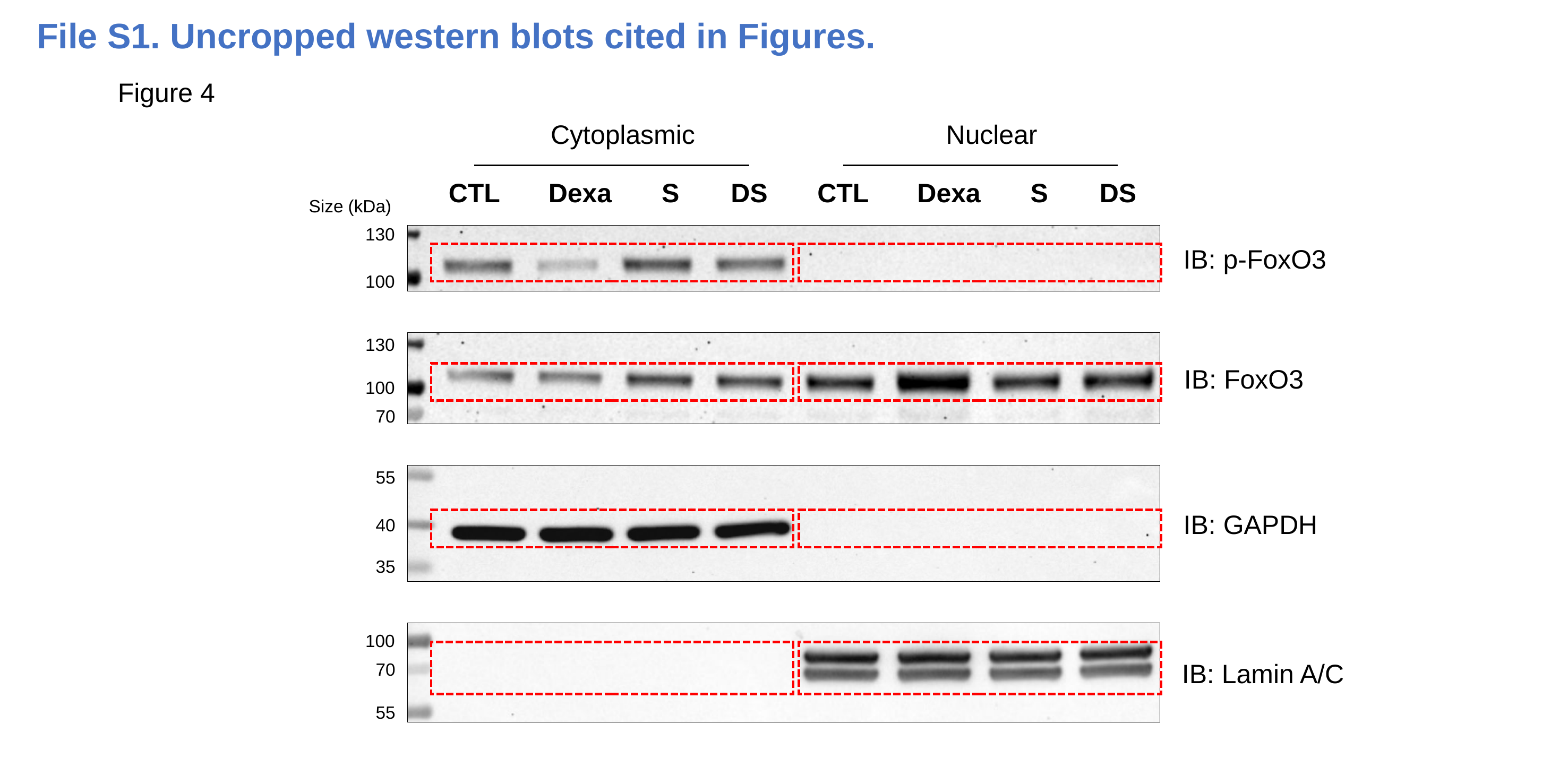

File S1. Uncropped western blots cited in Figures.
Figure 4
Cytoplasmic
Nuclear
CTL
Dexa
S
DS
CTL
Dexa
S
DS
Size (kDa)
130
IB: p-FoxO3
100
130
IB: FoxO3
100
70
55
IB: GAPDH
40
35
100
IB: Lamin A/C
70
55

## Slide 4
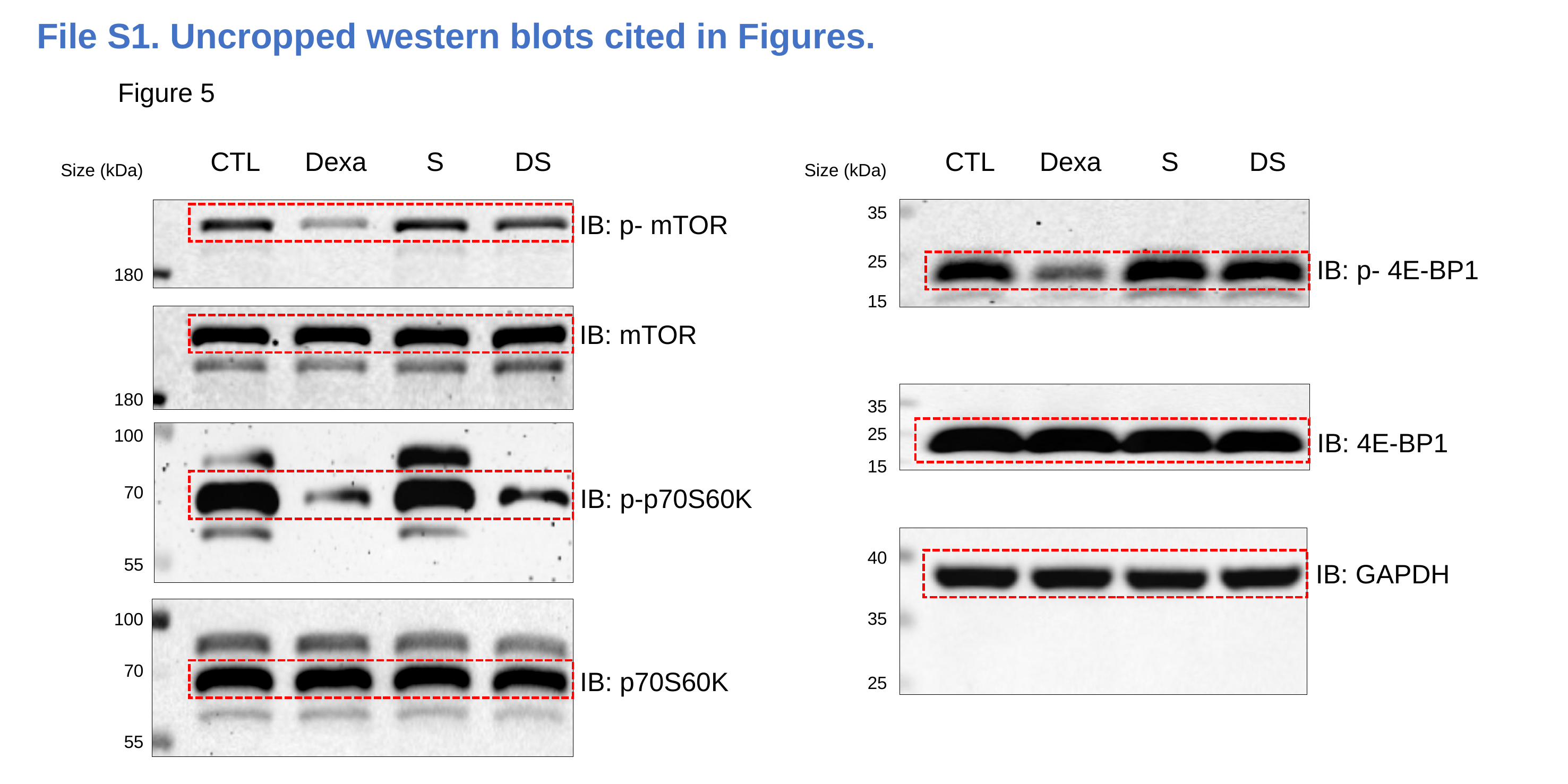

File S1. Uncropped western blots cited in Figures.
Figure 5
CTL
CTL
Dexa
S
DS
Dexa
S
DS
Size (kDa)
Size (kDa)
35
IB: p- mTOR
25
IB: p- 4E-BP1
180
15
IB: mTOR
180
35
25
100
IB: 4E-BP1
15
70
IB: p-p70S60K
40
55
IB: GAPDH
35
100
70
IB: p70S60K
25
55

## Slide 5
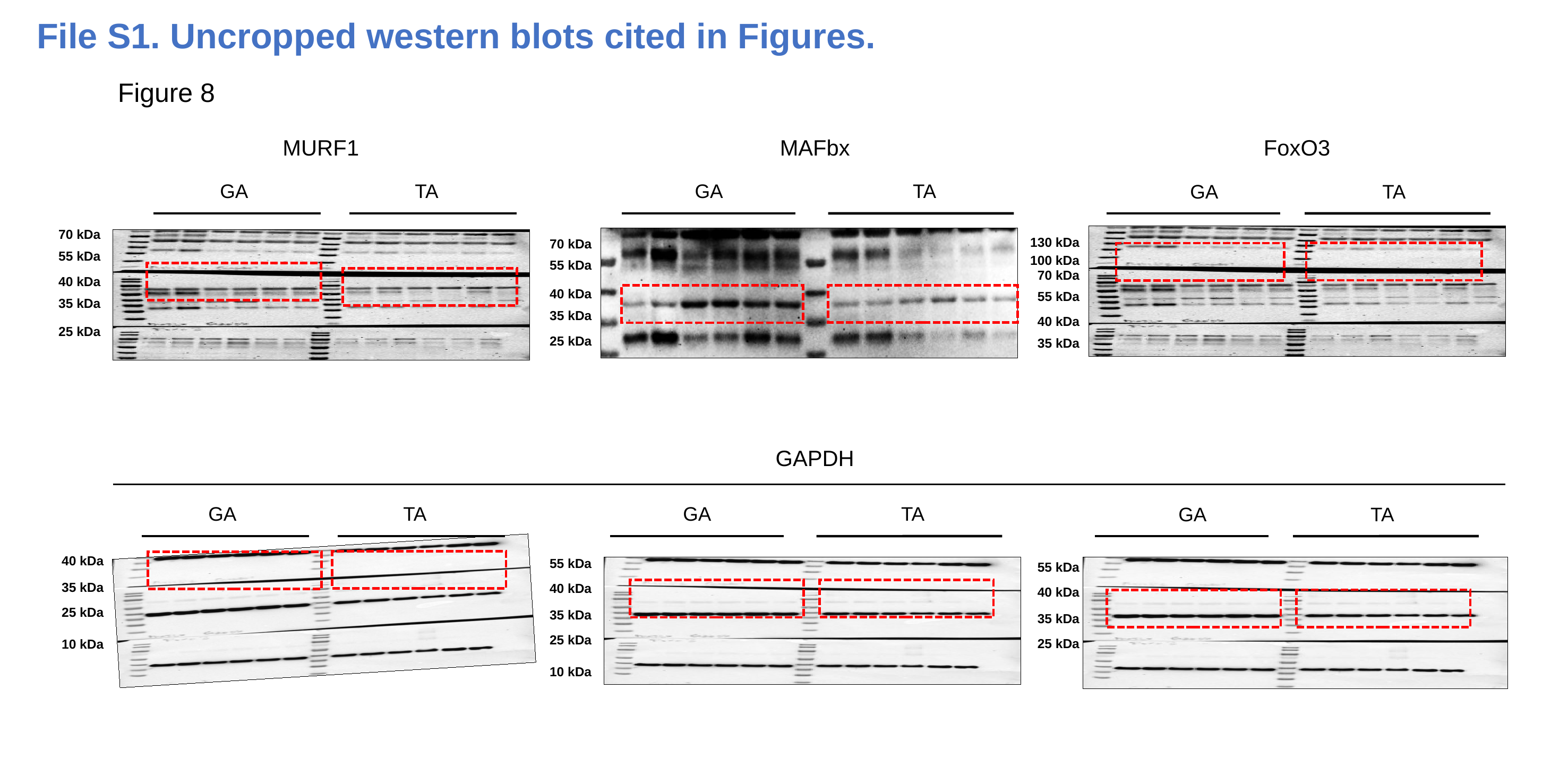

File S1. Uncropped western blots cited in Figures.
Figure 8
MURF1
MAFbx
FoxO3
GA
TA
GA
TA
TA
GA
70 kDa
130 kDa
70 kDa
55 kDa
100 kDa
55 kDa
70 kDa
40 kDa
40 kDa
55 kDa
35 kDa
35 kDa
40 kDa
25 kDa
25 kDa
35 kDa
GAPDH
GA
TA
GA
TA
TA
GA
40 kDa
55 kDa
55 kDa
35 kDa
40 kDa
40 kDa
25 kDa
35 kDa
35 kDa
25 kDa
25 kDa
10 kDa
10 kDa
